# mRNA sequencing of *Eucalyptus urograndis* trees supplemented with flavonoids shows changes on metabolic process and decrease of lignification

**DOI:** 10.1186/1753-6561-5-S7-P118

**Published:** 2011-09-13

**Authors:** Jorge Lepikson-Neto, Leandro Nascimento, Maria Carolina Scatollin, Wesley Marques, Marcela Salazar, Eduardo Camargo, Ramon Vidal, Danieli Gonçalves, Goncalo Pereira

**Affiliations:** 1 Laboratório de Genômica e Proteômica State University of Campinas-UNICAMP, Brazil; 2 Laboratório Nacional de Biociências – CNPEM/ABTLuS, Brazil

## Background

The flavonoids, naringenin-chalcone and narigenin, are intermediates in phenylpropanoid metabolism in plants, occupying the central position as primary intermediates in flavonoid biosynthesis and are synthesized by *chalcone synthase* (CHS) and *chalcone isomerase* (CHI) respectively.[1] It has been reported that supplementation of narigenin-chalcone and narigenin can inhibit the activity of 4CL in vitro, and suppress the growth and reduce lignin content in gramineous plants, while 4CL suppression affected plant phenotype and resulted on dwarfed trees on *Pinus*[2], and its down-regulation promoted enhanced growth phenotypes on transgenic aspens trees [3], also CHS expression controls flavonoid synthesis and reduced size phenotype on arabidopsis. [4] Eucalyptus is the main source of biomass for pulp and paper industries therefore it’s imperative to study the influence of flavonoid supplementation on Eucalyptus and what kind of overall impact it can have on plant development, especially wood formation and gene expression.

## Methods

### Plant Material

1 month *Eucalyptus* plantlets were divided into 3 groups with 25-30 individuals per group. Groups were given normal root induced nutritive solutions (Group 1) and added narigenin-chalcone (group 2) and narigenin (group 3). Treatment lasted 5 months with regular supplementation. Samples were then collected and properly stored.

### Histhochemical analyses

Stem samples were harvested and fixed in FAA for at least 24h. Sections were double stained with 1% alcoholic Safranin O and 1% aqueous Astra Blue. Hand sectioned tissue samples from the same material were stained with Mäule reagent: 1% KMnO4 for 15 min, 2% HCl for 5 min, and 2N NH4OH solution. Sections were observed with an Olympus BX51 microscope under white light, and the images were obtained with DP-72 digital camera and Image Pro Plus 6.3 software.

### mRNA Sequencing

RNA was extracted from xylem tissue according to [5] and mRNA libraries were prepared following instructions from the Illumina mRNA-Seq Prep Kit. Three distinct libraries were generated: *E.urograndis* plantets supplemented with narigenin-chalcone; narigenin; and control solution, containing 34,157,958 (65,75% mapped); 33,743,449 (64,80% mapped); 32,076,198 (65,09% mapped) reads, (reads size approximately 36pb).

### Assembling and annotation

We assembled 167,271 ESTs (130,290 from Genolyptus and 36,981 from NCBI) from several species and tissues using the program CAP3. [6] The assembly produced 53,412 unigenes (18,098 contigs and 35,314 singlets).

All unigenes were automatic annotated using the BLAST [7], including: non-redundant (NR) database of NCBI, pfam [8]. Moreover, we performed a functional annotation using the BLAST2GO software. [9]

The RNA-Seq reads were aligned against the assembled unigenes using the SOAP2 aligner [10] In order to perform the differential expression analysis between libraries, a normalization and statistical pipeline were applied using DEG-seq software [11] considering 99% of confidence rate (cut-off of 0.01).

## Results and discussion

Dual staining with Astra-blue and safranin-O histochemical analysis show, that both groups of plants treated with flavonoids exhibit a much greater blue stain, typical of celluloses content, when compared to control plants, while exhibiting a lesser amount of the red/purple stain related to phenolics compounds. The Mäule color reaction which provides an effective method for the detection of syringyl units, show an increase of S lignin content on both group of plants supplemented with narigenin and narigenin-chalcone. mRNA sequencing shows drastic changes on genes related with flavonoid sinhtesys, annotated contigs for those genes presented a much lower expression than control groups. Almost all genes from the phenylpropanoidpathway presented lower expression on treated groups, with genes at the beginning of the pathway presenting the most noteworthy reduction, while genes at the end of the pathway presented higher expression on treated groups which is interesting as it can suggest a shift from guaiacyl to syringyl monolignols increasing lignin solubility. Gene Onthology annotation shows significant differences among a number of metabolic process, including cell growth, cell division and response to stress. Among the most expressed genes were found heat-shock proteins, dehydrins and many no-hits. It is clear that supplementation with flavonoids alter gene expression especially regarding lignin biosynthesis.
